# Is a sense of coherence associated with prolonged grief, depression, and satisfaction with life after bereavement? A longitudinal study

**DOI:** 10.1002/cpp.2774

**Published:** 2022-08-16

**Authors:** Paul A. Boelen, Maja O'Connor

**Affiliations:** ^1^ Department of Clinical Psychology, Faculty of Social Sciences Utrecht University Utrecht The Netherlands; ^2^ ARQ National Psychotrauma Centre Diemen The Netherlands; ^3^ Unit for Bereavement Research, Department of Psychology Aarhus University Aarhus Denmark

**Keywords:** bereavement, depression, factorial validity, grief, sense of coherence

## Abstract

There is growing interest in psychological factors maintaining healthy functioning following adverse events. One such variable is a sense of coherence (SOC), an orientation to life comprising manageability, comprehensibility, and meaningfulness. Little research has examined the role of SOC in adjustment to bereavement. The present longitudinal study examined the role of SOC in recovery from loss, in a Danish sample (*N* = 221) of elderly spousally bereaved people. The aim was twofold. First, we aimed to establish the optimal measurement model of SOC, evaluating the fit of different factor solutions for the 29‐item SOC‐29 scale and 13‐item SOC‐13 scale, using confirmatory factor analysis. Second, we sought to examine associations of emerging SOC factors with symptoms levels of prolonged grief disorder (PGD) and depression, and with satisfaction with life, assessed concurrently (at 6 months post‐loss) and at two consecutive time points, 13 and 18 months post‐loss. Results showed that the three‐factor model of the SOC‐13 (with distinct manageability, comprehensibility, and meaningfulness factors) provided a good fit to our data. With respect to our second aim, analyses showed that the three SOC factors were associated with concurrently assessed PGD, depression, and satisfaction with life. In the analyses predicting outcomes at Wave 2 and Wave 3, meaningfulness (but not manageability and comprehensibility) predicted some of the outcomes, above and beyond baseline scores of the outcomes. Findings suggest that meaningfulness may increase healthy and attenuate unhealthy responses to loss. Helping bereaved people to experience life's demands as worthy of investment and engagement is likely an important target for bereavement care.

**Key Practitioner Message**

- Among elderly spousally bereaved people, a sense of coherence was found to encompass three distinguishable elements of manageability, comprehensibility, and meaningfulness.
- The three elements of a sense of coherence were associated with concurrently assessed reactions to loss.
- Baseline increased meaningfulness (but not manageability and comprehensibility) predicted decreased unhealthy and increased healthy bereavement‐responses later in time.
- Helping bereaved people to experience life's demands as worthy of investment and engagement and empowering them to identify resources needed to adjust are important targets for bereavement care.

## 1 INTRODUCTION

Research in the field of loss and trauma has strongly focused on identifying risk factors and psychological processes negatively influencing adjustment. Over the last years, there is growing interest in protective factors that maintain healthy functioning and boost adjustment following such events. One such variable is a sense of coherence (SOC), a concept central to the salutogenic theory of health (Antonovsky, 1979, 1987). Briefly, this theory postulates that health and disease are not dichotomous constructs. Instead, over the course of life, individuals move back and forth on a continuum between health and illness (or ease and disease), affected by positive and negative life experiences and resources available to deal with these experiences. SOC is one key concept within this framework and one key resource that confers protection to adversity. SOC is an orientation to life encompassing *manageability* (the sense of having capacities and resources to deal with challenges), *comprehensibility* (the perception that internal and external experiences and situations are understandable and can be structured), and *meaningfulness* (the basic sense that life's demands are worthy of investment and engagement). To measure SOC, Antonovsky (1979, 1987) developed the 29‐item SOC scale (SOC‐29) including items for all three theoretically derived SOC dimensions and a shortened version including a selection of 13 items (the SOC‐13). Both measures have been widely examined in different languages and samples. Studies have quite consistently supported the reliability and construct validity of the SOC‐13 and SOC‐29, but have left unclarity about the distinctiveness of the elements of manageability, comprehensibility, and meaningfulness (see, e.g., Antonovsky, 1993; Eriksson & Lindström, 2005; Eriksson & Mittelmark, 2016).

There is a growing evidence‐base supporting that elevated SOC is associated with increased positive outcomes, including general well‐being (Nilsson et al., 2010) and satisfaction with life (Moksnes et al., 2013) and decreased negative outcomes, including depression and anxiety (Flannery & Flannery, 1990). In addition, cross‐sectional research showed that increased SOC is associated with decreased symptom levels of posttraumatic stress disorder (PTSD) following different types of events (Schäfer et al., 2019). In longitudinal studies, people with higher SOC have been found to report lower posttraumatic stress following accidental injuries (Schnyder et al., 2008) and traumatic pregnancy loss (Engelhard et al., 2003).

It is conceivable that greater SOC may foster recovery from the death of a loved one. Manageability may help mourners to draw on available resources after their loss. Comprehensibility may fuel confidence that the consequences of the loss are understandable and controllable. Meaningfulness may strengthen the ability to commit to what is needed to adapt to the loss. To date, only few studies examined associations of SOC with psychological reactions to bereavement. Cross‐sectional research showed that higher SOC was associated with decreased grief among women who suffered perinatal bereavement (Uren & Wastell, 2002). In a heterogeneous sample of bereaved people, Bachem and Maercker (2016) examined associations of SOC with symptoms of prolonged grief disorder (PGD)—a disorder encompassing symptoms of separation distress and other intense grief reaction (e.g., difficulties accepting the loss, anger) present to a disabling and distressing degree (APA, 2022). They found increased SOC to be associated with lower symptom‐levels of PGD. Xiu et al. (2016) found similar associations between SOC and PGD in Swiss bereaved parents, but not Chinese bereaved parents. In people who lost loved ones to suicide, higher SOC scores were positively associated with healthy grief (assessed with the Texas Revised Inventory of Grief; Faschingbauer et al., 1977) and negatively with depression (Scharer & Hibberd, 2020); this suggests that greater SOC may increase healthy and attenuate unhealthy responses to loss. Wen et al. (2021) studied a large group of caregivers of deceased cancer patients from China and found elevated SOC at 1 month post‐loss to be associated with a trajectory of reactions characterized by lower depression and PGD severity.

There is still a need to further our understanding of SOC in recovery from loss. For instance, although different studies applied either the SOC‐13 or SOC‐29 to bereaved samples, no studies have systematically investigated the factor structure of these measures among mourners. It is, therefore, uncertain if, among people exposed to deaths of loved ones, SOC encompasses three distinguishable factors or can better be understood as a unitary concept. This is especially relevant considering that the factor structures of the SOC‐13 and SOC‐29 have been debated (Grevenstein et al., 2016; Grevenstein & Bluemke, 2021). Furthermore, to our knowledge, apart from Wen et al.'s (2021) study, no studies have examined the degree to which SOC is longitudinally associated with responses to bereavement. Moreover, it is important to establish whether their findings in a non‐western group generalize to western mourners. Relevant too is to examine associations of SOC with different responses to loss, including healthy (e.g., satisfaction with life) and unhealthy responses (e.g., PGD and depression). Given that SOC bears relevance to understanding individual differences in responses to bereavement and has hardly been studied, it is imperative to further knowledge about SOC in grief. Such knowledge may guide future interventions with bereaved people and enhance our general understanding of grief.

The current study used data from a Danish research project to expand knowledge on the role of SOC in recovery from loss. In that project, elderly spousally bereaved people completed self‐report measures at 2, 6, 13, 18, and 48 months post‐loss (O'Connor, 2010a, 2010b; O'Connor et al., 2015). The present study was based on data obtained at 6 months post‐loss (referred to as Wave 1, W1), 13 months post‐loss (Wave 2, W2), and 18 months post‐loss (Wave 3, W3). The study aim was twofold. First, we aimed to establish the optimal measurement of SOC from our data. Taking into account previous research investigating the factorial validity of SOC (e.g., Eriksson & Mittelmark, 2016), we compared different factor‐models of the SOC‐29 and the SOC‐13. Specifically, for the SOC‐29, we compared the fit of a one‐factor model and a three‐factor model, with items representing manageability, comprehensibility, and meaningfulness forming one or three factors, respectively. For the SOC‐13, we evaluated the fit of a one‐factor model, a three‐factor model (with distinct factors for manageability, comprehensibility, and meaningfulness), plus a two‐factor model, with items for manageability and comprehensibility forming one factor. Grevenstein et al. (2016) found the two‐factor model of the SOC‐13 to explain more variance in psychological distress than one‐factor and three‐factor models; this accords with the notion that meaningfulness is a distinct, more basic element of SOC, driving the motivation to mobilize resources to comprehend and manage life stressors. Considering that all three models have yielded at least some support in prior research (Grevenstein & Bluemke, 2021), we decided to compare their fit in the present work.

Our second aim was to examine associations of emerging SOC factors with indices of PGD, depression, and satisfaction with life, assessed concurrently (at 6 months post‐loss, W1) and the association of SOC at W1 with these outcomes at W2 and W3 (13 and 18 months post‐loss). We focused on negative and positive outcomes, considering that adjustment to loss involves both mitigating negative emotions and maintaining positive psychological functioning. Assuming that the analyses would show that, in our sample, SOC consists of multiple, distinguishable components, we intended to investigate both the association of the distinct components with the outcomes as well as the association of the SOC factors with outcomes when controlling for the shared variance between SOC factors. Considering the lack of knowledge on the role of SOC in recovery from loss, examining both the distinct and combined impact of SOC factors on bereavement outcomes was deemed both clinically and theoretically relevant, by elucidating potential underlying mechanisms of problems in recovery from loss and targets for interventions.

We had no hypotheses about the factor structure of the SOC items, considering that prior work has supported different factorial structures. With respect to our second aim, we anticipated that SOC would be negatively related with PGD and depression symptoms and positively with satisfaction with life. Again, because of the lack of research in bereaved samples, we had no specific hypotheses about the strengths of these relationships nor about the relative importance of SOC factors emerging from our analyses.

## 2 METHODS

### 2.1 Participants and procedure

Of 296 participants included at the 2‐month assessment, in the current study, data from *N* = 221, *N* = 176, and *N* = 169 were available at 6 months (W1), 13 months (W2), and 18 months post‐loss (W3), respectively. In the W1 sample (*N* = 221), participants had a mean age of 72.19 (SD = 4.23) years: 130 (58.8%) were female, 88 (39.8%) were male (gender was unknown for *n* = 3). They had a mean of 8.19 years of public schooling (SD = 1.62; range 5–14, *n* = 12 missing). All had lost a spouse or partner. As noted, for all participants, the time elapsed since the loss was 6 months (at W1). Participants had cohabited with their partner 45.34 (SD = 10.73) years before the partner's death, on average. They had 2.61 (SD = 1.51, range 0–9, *n* = 6 missing) children on average; *n* = 190 (86%, n = 9 missing) had faced a period of the spouse's illness before the partner died. The self‐reported cause of death was cancer in 91 (41.2%) cases, cardio vascular disease in 34 (15.4%) cases, dementia in five (2.3%) cases, an accident in three (1.4%) cases, diabetes in three (1.4%) cases, other causes (sometimes including combined causes, e.g., diabetes and cardio vascular) in 74 (33.5%) cases, and unknown in 11 (5%) cases.

### 2.2 Measures

Data were gathered using self‐report questionnaires. The first part contained questions on socio‐demographics and loss‐related variables. The second part included different standardized questionnaires measuring PGD symptoms, depression symptoms, satisfaction with life, and SOC. As described elsewhere (e.g., O'Connor, 2010a, 2010b; O'Connor et al., 2015), Danish versions of non‐Danish questionnaires were developed based on formal forward‐backward translation procedures. Alpha reliabilities of these scales are all shown in Table 1.

**TABLE 1:** Zero order correlations, means, and standard deviations

Measure	1	2	3	4	5	6	7	8	9	10	11	*N*	M	SD	Range	Alpha reliability
1. W1 comprehensibility	‐											218	24.26	5.07	5–35	.70
2. W1 manageability	.68**											219	21.44	3.68	4–28	.59
3. W1 meaningfulness	.60**	.50**										219	22.41	4.01	6–28	.74
4. W1 PG	−.32**	−.21**	−.35**									200	32.93	10.72	17–75	.91
5. W1 depression	−.58**	−.43**	−.59**	.65**								214	8.34	6.20	0–41	.86
6. W1 SWL	.46**	.39**	.64**	−.36**	−.52**							215	25.59	5.79	8–35	.86
7. W2 PG	−.26**	−.22**	−.31**	.87**	.56**	−.28**						165	31.64	10.98	15–70	.92
8. W2 depression	−.46**	−.36**	−.47**	.48**	.77**	−.40**	.59**					173	8.40	6.61	0–37	.88
9. W2 SWL	.45**	.32**	.60**	−.31**	−.48**	.71**	−.33**	−.51**				171	25.92	5.49	11–35	.88
10. W3 PG	−.17*	−.18**	−.29**	.83**	.54**	−.22**	.87**	.53**	−.29**			163	30.10	10.59	15–75	.91
11. W3 depression	−.45**	−.33**	−.48**	.60**	.77**	−.39**	.58**	.80**	−.44**	.59**		167	7.79	6.61	0–28	.89
12. W3 SWL	.43**	.35**	.62**	−.35**	−.52**	.69**	−.32**	−.51**	.75**	−.35**	−.56**	167	26.08	5.71	12–35	.87

*Note*: Comprehensibility, manageability, and meaningfulness assessed with the SOC‐13. Prolonged grief was measured with the Inventory of Complicated Grief. Depression was measured with the Beck Depression Inventory. Satisfaction with life was measured with the Satisfaction with Life scale.

Abbreviations: PG, prolonged grief; SWL, satisfaction with life; W1, Wave 1; W2, Wave 2; W3, Wave 3.

* *p* < .05.

** *p* < .01.

*** *p* < .001.

An adapted version of the Inventory of Complicated Grief (Prigerson et al., 1995) was used to measure PGD symptoms. It includes 15 symptoms of PGD and other putative markers of disordered grief. Participants rated the frequency of symptoms in the prior month, on 5‐point Likert scales with anchors 1 = *none/almost never* to 5 = *a lot/all the time*. The summed score was used as an index of PGD symptom severity. The ICG has sound psychometric properties (Prigerson et al., 1995).^1^

The Beck Depression Inventory (BDI; Beck et al., 1988) was used to measure depression. It includes 21 sets of four statements representing depressive symptoms at increasing levels of severity. Statements are rated on a 0–3 scale (with higher scores representing more severe depression) and summed to get an index of depression. The measure has strong psychometric properties (Beck et al., 1988).

The Satisfaction with Life Scale is a five‐item measure that was constructed by Diener et al. (1985) to measure the global judgement about one's life satisfaction. Participants are asked to rate how much they agree with items, on a 7‐point scale, with anchors 1 = *strongly disagree* to 7 = *strongly agree*. The measure has good psychometric properties.

The SOC‐29 was used to measure three domains of comprehensibility, manageability, and meaningfulness. The SOC‐29 includes all 13 items from the SOC‐13. Both versions were originally developed by Antonovsky (1987). Items are scaled along a dimensional scale, ranging from 1 to 7, with anchoring phrases at both extremes. Items of the SOC‐13 are shown in Table S1. An example of an item on meaningfulness is “Do you have the feeling that you don't really care about what goes on around you?” rated on a scale from 1 = *very seldom or never* to 7 = *very often* (with scores being reversed before being summed with other item scores). As noted above, evidence supported psychometric properties of the SOC‐29 and SOC‐13 but the factor structure of both scales has been debated (Eriksson & Mittelmark, 2016). One‐factor, two‐factor, and three‐factor models evaluated in this study have all received support in different studies (Grevenstein & Bleumke, 2017, 2021).

### 2.3 Statistical analyses

Our statistical analyses included four consecutive steps. First, to address our first aim, we examined the factor structure of SOC items using confirmatory factor analysis (CFA) implemented in MPlus (Muthén & Muthén, 1998‐2012). Using data from the W1 sample (*N* = 221), we successively evaluated the fit of a one‐factor solution and three‐factor solution of the SOC‐29 and one‐factor, two‐factor, and three‐factor solutions of the SOC‐13. (For the SOC‐29, we did not evaluate a two‐factor solution because, to our knowledge, that model has not been validated in prior research.) As estimation method, maximum likelihood with robust standard errors (MLR) was used. To evaluate model fit, we considered the comparative fit index (CFI) and Tucker–Lewis index (TLI) with values >.90 indicating adequate fit, and the root‐mean‐square error of approximation (RMSEA) and standardized root mean square residual (SRMR) with values <.08 indicating acceptable fit (Hu & Bentler, 1999). To compare the fit of the models, we considered the Akaike information criterion (AIC), Bayesian information criterion (BIC), and sample size adjusted Bayesian information criterion (SS‐BIC). We used χ^2^ difference tests to test the differences between nested models, using the Satorra‐Bentler scaling correction (Satorra & Bentler, 2001).

As a second step, we calculated zero‐order correlations between indices of SOC (at W1) and PGD, depression, and functional impairment (at W1–W3). In addition, we examined whether scores on these indices differed as a function of the socio‐demographic and loss‐related variables we assessed, using analysis of variance and Pearson correlations. As a third step, we examined whether participants who discontinued participation at W2 differed from those continuing participation in terms of all variables assessed at W1 using analysis of variance for continues variables and Fisher's exact tests for dichotomous variables. Similar analyses were done to compare people who continued vs. discontinued participation at W3.

As a fourth step and to address our second aim, we performed a series of multiple regression analyses to examine associations of emerging SOC factors with symptom‐levels of PGD and depression, and satisfaction with life, at W1, W2, and W3. In examining concurrent associations, we planned to examine the association of each distinct emerging SOC factor with the outcomes (PGD, depression, satisfaction with life); next, we planned to examine associations of emerging SOC factors with the outcomes, while controlling for the shared variance between the SOC factors. In examining associations of SOC factors with outcomes at W2 and W3, a similar procedure was used, albeit that W1 scores of the outcome variables were controlled in the analyses, to examine if SOC factors predicted outcomes at W2 and W3, beyond outcomes at W1. There were occasional missing values on some items. For aim 1, full information maximum likelihood was used to deal with these missings. For the analyses that included the measures of SOC, PGD, depression, and functional impairment, listwise deletion was employed when participants had >20% missing variables on the items of the measure under consideration and the remaining occasional missing variables of questionnaire items were handled using mean imputation. This resulted in slightly varying sample sizes across analyses.

## 3 RESULTS

### 3.1 Factor structure of the SOC items

Fit indices for the models that we tested are shown in Table 2. For the SOC‐29, the one‐factor and three‐factor models did not fit the data (e.g., CFI and TLI were too low). Modification indices of the three‐factor model pointed at correlations between one of the manageability items (Has it happened that people whom you counted on disappointed you?) and one of the comprehensibility items (Has it happened in the past that you were surprised by the behaviour of people whom you thought you knew well?). This potentially stemmed from content overlap, considering that both items represented a critical view on other people. However, an adjusted three‐factor model, allowing the error‐terms of this pair to be correlated, still did not yield a good fit.

**TABLE 2:** Fit indices factor models sense of coherence items

	Chi square	DF	AIC	BIC	SS‐BIC	RMSEA (90% CI)	CFI	TLI	SRMR
29 items version									
1‐factor model	845.54	377	20,342.82	20,638.40	20,362.75	0.075 (0.068–0.082)	0.761	0.743	0.078
3‐factor model	787.16	374	20,279.90	20,585.74	20,300.52	0.071 (0.064–0.078)	0.790	0.772	0.077
3‐factor model, with correlated errors^a^	706.38	373	20,187.22	20,496.46	20,208.08	0.064 (0.056–0.071)	0.830	0.815	0.073
13 items version									
1‐factor model	209.55	65	9274.784	9407.36	9283.77	0.100 (0.085–0.116)	0.763	0.715	0.077
2‐factor model	182.69	64	9244.43	9380.35	9253.59	0.092 (0.076–0.107)	0.805	0.763	0.071
2‐factor model correlated errors^b^	105.22	62	9157.43	9300.15	9167.05	0.056 (0.037–0.074)	0.929	0.911	0.055
3‐factor model	182.31	62	9245.46	9388.19	9255.09	0.094 (0.078–0.110)	0.803	0.752	0.071
3‐factor model, correlated errors^b^	101.93	60	9156.51	9306.03	9166.59	0.056 (0.037–0.075)	0.931	0.911	0.054

Abbreviations: AIC, Akaike information criterion; BIC, Bayesian information criterion; CFI, comparative fit index; DF, degrees of freedom; RMSEA, root‐mean‐square error of approximation; SRMR, standardized root mean square residual; SS‐BIC, sample size adjusted Bayesian information criterion; TLI, Tucker–Lewis index.

^a^ Error terms of one manageability item (Has it happened that people whom you counted on disappointed you?) and one comprehensibility item (Has it happened in the past that you were surprised by the behaviour of people whom you thought you knew well?) were correlated.

^b^ Error terms of the same manageability item and comprehensibility item were correlated; error terms of one manageability item (Many people—even those with a strong character—sometimes feel like sad sacks [losers] in certain situations. How often have you felt this way in the past?) and one comprehensibility item (When something happened, have you generally found that: [you overestimated or underestimated its importance – you saw things in the right proportion]) were also correlated.

For items included in the SOC‐13, the one‐factor model did not fit well. The two‐factor model fit the data better than the one‐factor model (∆*χ* ^2^ = 22.15, ∆*df* = 1, *p* < .001), but also did not provide good fit estimates (e.g., CFI and TLI were too low, RMSEA was too high). Modification indices pointed out that errors of the same two items that had correlated errors in the SOC‐29 were correlated in the SOC‐13. In addition, errors terms of one of the manageability items (Have you felt like a loser?) and one of the comprehensibility (Have you overestimated/underestimated the importance of things happening?) were also correlated; this was also potentially due to content overlap, considering that these items both represented critical self‐evaluation. The adjusted two‐factor model, with correlations between these two pairs of error terms fit better than the one‐factor model (∆*χ* ^2^ = 93.20, ∆*df* = 3, *p* < .001) and the two‐factor model (∆*χ* ^2^ = 72.52, ∆*df* = 2, *p* < .001) and had good fit estimates (Table 2).

The SOC‐13 three‐factor model fit the data better than the one‐factor model (∆*χ* ^2^ = 22.79, ∆*df* = 3, *p* < .001), but did not have good fit estimates. Again, modification indices pointed at correlations between the errors of the two aforementioned item pairs. The adjusted three‐factor model, with correlations between these error terms, fit better than the one‐factor model (∆*χ* ^2^ = 93.43, ∆*df* = 5, *p* < .001) and the three‐factor model (∆*χ* ^2^ = 84.03, ∆*df* = 2, *p* < .001) and had good fit estimates (Table 2).

The two‐factor model with correlated errors and the three‐factor model with correlated errors had similar fit estimates and—accordingly—the *χ* ^2^ difference test was not significant (∆*χ* ^2^ = 3.39, ∆*df* = 2, *p* = .184). Because research on SOC in grief is sparse, it was deemed informative to retain the three‐factor model, distinguishing the three factors put forth by Antonovsky (1979), in subsequent analyses. Items and factor loading of the SOC‐13 are shown in Table S1. Correlations of the comprehensibility factor with the manageability and meaningfulness factors were .91 and .88, respectively; the correlation between the manageability and meaningfulness factors was .72 (*p*s < .001).

### 3.2 Descriptive data and correlations between variables

Zero‐order correlations between the three SOC factors at W1 and PGD severity, depression severity, and satisfaction with life at W1, W2, and W3 are shown in Table 1, together with mean scores on the variables. Correlation between the SOC factors were strong. Across W1–W3, correlations of SOC factors with PGD were small to moderate and correlations with depression and satisfaction with life were moderate to strong. For exploratory reasons, we examined if scores on the SOC factors at W1 and PGD, depression, and satisfaction with life at W1–W3 (shown in Table 1), differed as a function of the socio‐demographic and loss‐related variables we assessed. These variables included gender, age, number of years in public school, time of cohabiting with the partner, number of children, whether a period of illness preceded the death (yes/no), and cause of death—dichotomized into cancer deaths vs. other deaths. It was found that whether a period of illness preceded the death (yes/no) was associated with PGD at W1: significantly higher scores were reported when there was no period of illness, M = 37.16, SD = 11.64 vs. M = 32.10, SD = 10.38, *F*(1, 190) = 3.96, *p* = .048. This variable was also associated with satisfaction with life scores at W1, W2, and W3: significantly higher satisfaction with life scores were reported when a period of illness preceded the death, W1: M = 26.03, SD = 5.63 vs. M = 22.52, SD = 5.92, *F*(1, 205) = 7.25; W2: M = 26.32, SD = 5.35 vs. M = 22.85, SD = 5.69, *F*(1, 165) = 5.96; W3: M = 26.49, SD = 5.69 vs. M = 23.55, SD = 5.56, *F*(1, 161) = 4.27; all *p*s < .05. Moreover, dichotomized cause of death was associated with PGD severity at W1, W2, and W3, with significantly higher scores reported by those who lost a spouse to cancer, W1: M = 34.67, SD = 10.41 vs. M = 31.63, SD = 10.81, *F*(1, 199) = 3.97; W2: M = 34.18, SD = 10.14 vs. M = 29.86, SD = 11.24, *F*(1, 164) = 6.38; W3: M = 32.61, SD = 10.75 vs. M = 28.35, SD = 10.18, *F*(1, 162) = 6.59, all *p*s < .05. Finally, having more children was associated with stronger satisfaction with life at W2 (r = .15, *p* = .03).

### 3.3 Differences between people continuing versus discontinuing participation

Of all 221 participants at Wave 1, the 176 who still participated at Wave 2 did not differ from the 45 discontinuing their participation on any of the variables assessed at Wave 1 (all socio‐demographic and loss‐related measures, the SOC factors and PGD, depression, and functional impairment). Of all participants at Wave 1, the 169 people who still participated at Wave 3 did not differ from the 52 discontinuing their participation on all variables assessed at Wave 1.

### 3.4 Associations of SOC factors with PGD, depression, and satisfaction with life at W1‐W3

Table 3 summarizes outcomes of the regression analyses regarding the concurrent associations between SOC and outcomes. All three SOC factors were inversely associated with PGD severity in distinct analyses (Models 1–3). Comprehensibility and meaningfulness were significantly correlated with W1 PGD when all SOC factors were considered simultaneously (Model 4). Similar outcomes were found in the regression analyses with depression severity as dependent variable. As for satisfaction with life, all three SOC factors were correlated with satisfaction with life in distinct analyses (Models 1–3); meaningfulness was the only SOC factor associated with this outcome, when all factors were considered simultaneously (Model 4).

**TABLE 3:** Summary of regression analyses with comprehensibility, manageability, and meaningfulness predicting concurrent prolonged grief, depression, and satisfaction with life

	Model 1	Model 2	Model 3	Model 4
DV = prolonged grief at wave 1				
IV = comprehensibility (Beta)	−.31***	−		−.19*
Manageability (Beta)	−	−.21**	−	−.04
Meaningfulness (Beta)	−	−	−.35***	−.27**
*F*	21.91	9.28	28.04	11.55
DF	1, 196	1, 196	1, 196	3, 192
*p*	<.001	<.001	<.001	<.001
Adjusted *R* ^2^	.096	.040	.121	.140
DV = depression at wave 1				
IV = comprehensibility (Beta)	−.58***	−	−	−.33***
Manageability (Beta)	−	−.43***	−	−.05
Meaningfulness (Beta)	−	−	−.59***	−.37***
*F*	105.11	46.78	111.71	53.52
DF	1, 210	1, 210	1, 210	3, 206
*p*	<.001	<.001	<.001	<.001
Adjusted *R* ^2^	.330	.178	.344	.430
DV = satisfaction with life at wave 1				
IV = comprehensibility (Beta)	.46***	−	−	.07
Manageability (Beta)	−	.39***	−	.08
Meaningfulness (Beta)	−	−	.64***	.55***
*F*	56.92	38.45	149.76	52.42
DF	1, 210	1, 211	1, 211	3, 206
*p*	<.001	<.001	<.001	<.001
Adjusted *R* ^2^	.210	.150	.412	.425

Abbreviations: DV, dependent variable; IV, independent variable.

* *p* < .05.

** *p* < .01.

*** *p* < .001.

Table 4 summarizes the regression analyses with outcomes at W2 as dependent variables and SOC factors at W1 and outcomes at W1 as predictor variables. Neither in distinct models, nor combined, did SOC factors predict PGD at W2, beyond PGD at W1, or depression at W2 beyond depression at W1. As for satisfaction with life, comprehensibility and meaningfulness predicted W2 satisfaction with life in distinct analyses (Models 1 and 3); meaningfulness predicted W2 satisfaction with life beyond W1 satisfaction with life when controlling for the shared variance between SOC factors (Model 4).

**TABLE 4:** Summary of regression analyses with comprehensibility, manageability, and meaningfulness predicting prolonged grief, depression, and satisfaction with life at W2

	Model 1	Model 2	Model 3	Model 4
DV = W2 prolonged grief				
IV = W1 prolonged grief (Beta)	.87***	.87***	.85***	.86***
Comprehensibility (Beta)	<.01	−	−	.04
Manageability (Beta)	−	< .01	−	.02
Meaningfulness (Beta)	−	−	−.05	−.08
*F*	243.08	239.31	236.66	116.12
DF	2, 152	2, 151	2, 151	4, 148
*p*	<.001	<.001	<.001	<.001
Adjusted *R* ^2^	.762	.757	.755	.752
DV = W2 depression				
IV = W1 depression (Beta)	.73***	.75***	.70***	.68***
Comprehensibility (Beta)	−.07	−	−	−.06
Manageability (Beta)	−	−.02	−	.04
Meaningfulness (Beta)	−	−	−.11^†^	−.09
*F*	117.90	114.95	114.19	56.39
DF	2, 165	2, 164	2, 164	4, 161
*p*	<.001	<.001	<.001	<.001
Adjusted *R* ^2^	.583	.584	.577	.573
DV = W2 satisfaction with life				
IV = W1 satisfaction with life (Beta)	.62***	.66***	.56***	.54***
Comprehensibility (Beta)	.18**	−	−	.13^†^
Manageability (Beta)	−	.11^†^	−	−.06
Meaningfulness (Beta)	−	−	.24***	.21*
*F*	89.62	83.25	100.41	50.75
DF	2, 164	2, 163	2, 163	4, 160
*p*	<.001	<.001	<.001	<.001
Adjusted *R* ^2^	.516	.499	.546	.548

Abbreviations: DV, dependent variable; IV, independent variable; W1, Wave 1; W2, Wave 2.

^†^ *p* < .10.

* *p* < .05.

** *p* < .01.

*** *p* < .001.

Table 5 summarizes the regression analyses with outcomes at W3 as dependent variables and SOC factors at W1 and outcomes at W1 as predictor variables. The SOC factors did not predict PGD severity at W3, neither when considered separately (Models 1–3) nor together (Model 4). Lower meaningfulness at W1 predicted increased depression at W3, beyond W1 depression, when considered separately (Model 3) and together with the other SOC factors (Model 4). In the regression analysis predicting W3 satisfaction with life, we found that stronger comprehensibility (in Model 1), manageability (Model 2), and meaninglessness (Model 3) at W1 predicted increased satisfaction with life at W3, while controlling W1 satisfaction with life. When controlling for the shared variance between the SOC factors (Model 4), increased meaningfulness was the only significant predictors of W3 satisfaction with life.

**TABLE 5:** Summary of regression analyses with sense of coherence predicting prolonged grief, depression, and satisfaction with life at Wave 3

	Model 1	Model 2	Model 3	Model 4
DV = W3 prolonged grief				
IV = W1 prolonged grief (Beta)	.83***	.83***	.80***	.81***
Comprehensibility (Beta)	.02	−	−	.04
Manageability (Beta)	−	−.04	−	−.07
Meaningfulness (Beta)	−	−	−.05	−.10^†^
*F*	161.75	159.87	156.87	78.32
DF	2, 152	2, 151	2, 151	4, 148
*p*	<.001	<.001	<.001	<.001
Adjusted *R* ^2^	.676	.675	.671	.670
DV = W3 depression				
IV = W1 depression (Beta)	.72***	.76***	.69***	.69***
Comprehensibility (Beta)	−.08	−	−	−.08
Manageability (Beta)	−	<−.01	−	−.07
Meaningfulness (Beta)	−	−	−.13*	−.12*
*F*	116.02	112.23	116.33	57.98
DF	2, 159	2, 158	2, 160	4, 155
*p*	<.001	<.001	<.001	<.001
Adjusted *R* ^2^	.588	.582	.590	.589
DV = W3 satisfaction with life				
IV = W1 satisfaction with life (Beta)	.61***	.64***	.53***	.51***
Comprehensibility (Beta)	.16**	−	−	.06
Manageability (Beta)	−	.14*	−	<−.01
Meaningfulness (Beta)	−	−	.28***	.26***
*F*	78.82	74.75	97.06	47.95
DF	2, 158	2, 157	2, 157	4, 154
*p*	<.001	<.001	<.001	<.001
Adjusted *R* ^2^	.493	.488	.547	.543

Abbreviations: DV, dependent variable; IV, independent variable; W1, Wave 1; W3, Wave 3.

^†^ *p* < .10.

* *p* < .05.

** *p* < .01.

*** *p* < .001.

### 3.5 Additional regression analyses

We examined if the outcomes of the regressions summarized in Tables 2, 3, 4 changed when we controlled for the three loss‐related variables that were associated with some of the outcomes, namely: number of children, dichotomized cause (cancer vs. other cause), and having had a period of illness before the death (yes/no). There were two minor changes: The association of comprehensibility with PGD at W1 was no longer significant in the model that included all three SOC factors (Model 4; β = −.15, *p* = .14). The association of meaningfulness with W3 PGD, controlling W1 PGD at the other SOC factors, passed the threshold for significance (β = −.14, *p* < .05). All other significant findings remained significant and all other nonsignificant findings remained nonsignificant. Thus, associations of SOC factors with outcomes reported in Tables 2, 3, 4 were not moderated by the three loss‐related variables associated with some of the outcomes (number of children, dichotomized cause of loss, and a period of illness preceding the death).

As noted, the two‐factor model (with one factor including the comprehensibility and manageability items and a second factor spanning meaningfulness items) fit the data equally well as the three‐factor model (Table 2). For exploratory reasons, we reran all the regression analyses predicting PGD, depression, and satisfaction with life, but instead of including the three SOC factors, we now included two SOC factors (factor 1: comprehensibility/manageability, factor 2: meaningfulness). Outcomes are summarized in Tables S2–S4. Outcomes were generally consistent with outcomes of the regressions with the three SOC factors. In all analyses where the two distinguished comprehensibility and manageability factors were significantly associated with the dependent variable (e.g., satisfaction with life at W2 and W3), the combined factor (spanning comprehensibility and manageability items) predicted the outcome as well. In all analyses where the distinct factors did not predict outcomes, the combined also did not. So, it appears that combining the manageability and comprehensibility items did not enhance explanatory power of the analyses.

## 4 DISCUSSION

A gradually growing evidence base shows that a sense of coherence (SOC), encompassing a sense that one's internal and external world are comprehensible, manageable, and meaningful, offers protection against the negative impact of stressful life events (Engelhard et al., 2003; Schäfer et al., 2019; Schnyder et al., 2008). So far, very few studies have examined the role of SOC in responses to bereavement. The current study used data from a large Danish multi‐wave study, among elderly spousally bereaved individuals (e.g., O'Connor, 2010a, 2010b; O'Connor et al., 2015), to further knowledge about this issue.

A first aim was to identify the optimal measurement of SOC with the data available for the current investigation. Therefore, we first examined the factor structure of different measurement models of the SOC‐29 and the SOC‐13. For the SOC‐29, one‐factor and three‐factor models did not fit the data well. A three‐factor model with correlated errors of two similarly worded items approached (but did not pass) the threshold for acceptable model fit. In our examination of the factor structure of the SOC‐13, the two‐factor and three‐factor models fit the data better than the one‐factor model but did not pass the threshold for good fit. Modification indices revealed that error terms of two items referring to critical views on other people and two other items referring to a critical self‐evaluation were correlated. When allowing residual correlations for these two item‐pairs, both the two‐factor and three‐factor models fit the data well; the fit of these models did not differ significantly.

The findings indicate that, in our sample, the more parsimonious set of 13 items yielded a better measurement of SOC than the combination of 29 items. Our findings are generally consistent with research findings showing that the two‐factor model represents a good model of the SOC‐13 (Grevenstein & Bluemke, 2021) and other studies supporting the three‐factor structure (see Bonacchi et al., 2012). That said, some studies have shown that the scale is adequately represented by a one‐factor model (e.g., Antonovsky, 1993) or alternative three‐factor models (e.g., Bachem & Maercker, 2016). Because studies on the factor structure of SOC items have relied on many different samples, it is not clear whether and how exactly this factor structure differs between different groups of people. It is, therefore, also hard to speculate whether some of our results, including the finding that one‐factor and three‐factor solutions of the SOC‐29 did not fit our data, are due to specific characteristics of our sample. It remains an important issue for future research to investigate if, to what extent, and for what reason the structure of SOC items varies between different groups.

Considering that Antonovsky (1979) originally meant to distinguish three elements and that the three‐factor model for the SOC‐13 fit well, we included these factors in the regression analyses we performed to achieve our second aim which was to examine associations of SOC with concurrently and longitudinally assessed bereavement outcomes. Findings showed that all three SOC factors were significantly correlated with PGD, depression, and satisfaction with life when considered in distinct analyses (Table 3). When controlling for the shared variance between the SOC factors, comprehensibility and meaningfulness both explained unique variance in depression and PGD, whereas meaningfulness was significantly associated with satisfaction with life.

The importance of meaningfulness in affecting responses to loss was also clear when considering the longitudinal analyses. SOC factors were unrelated to PGD severity at W2 and W3, when controlling W1 PGD albeit that the association of meaningfulness with PGD at W3 trended toward significance in the regression including all three SOC factors. SOC factors did not predict W2 depression beyond W1 depression. Meaningfulness predicted W3 depression, beyond W1 depression, when considered alone and together with the other SOC factors. When considered in distinct analyses, the three SOC factors were associated with W2 and W3 satisfaction with life, when controlling W1 satisfaction with life. Meaningfulness was the only SOC factor predicting satisfaction with life at W2 and W3 when all three SOC factors were considered together.

Considering these findings, the following stands out. Meaningfulness consistently turned out to be the most important element of SOC in predicting consequences of bereavement. This is consistent with Antonovsky's (1987) original notion that meaningfulness has the strongest bearing on people's abilities to manage adversities. Apparently, the sense that consequences of one's loss‐experience are challenges that are worthy to investigate time and energy in—implicated in elevated meaningfulness—has an impact on adjustment to loss. The sense that one's external and internal worlds are understandable and that one has the resources needed to manage the consequences of the loss—implicated in elevated comprehensibility and manageability—are seemingly less important. The findings are reminiscent of prior theorizing and research pointing at the importance of meaning attribution and meaning making in grief (Neimeyer et al., 2014; Smid, 2020). The present findings add to this prior work by showing that, apart from the ability to assign meaning to a specific loss‐experience, the broader life orientation that *stressors are worth facing* attenuates the emotional pain of bereavement. Notable too is that increased meaningfulness was associated with increased satisfaction with life and, albeit to a lower degree, decreased levels of PGD and depression. This is consistent with prior theorizing and research indicating that elevated SOC both acts as buffer between negative events and distress and directly inflates positive outcomes in the face of these events (Schäfer et al., 2019). Following spousal loss, higher meaningfulness likely encourages people to continue investing energy in activities that were fulfilling before the loss and explore options for new activities. This type of behaviour is likely to attenuate sadness, yearning, and other grief reactions and to maintain satisfaction with the life.

Strengths of this study include its longitudinal design, multiple follow‐up assessments, and relatively homogeneous sample. The study also has some limitations. First, the use of self‐report measures to assess all the variables may have inflated associations between variables because of shared method variance. Second, although the ICG is still commonly used, it was constructed way before recent conceptualizations of PGD emerged, including those now included in DSM‐5‐TR (APA, 2022) and ICD‐11 (WHO, 2019). Hence, additional research should focus on these more recent descriptions of disordered grief. Third, we were primarily interested in the potential impact of SOC on bereavement outcome and did not examine possible moderators and mediators. Future studies are needed to explore if access to social support, adaptive cognitive behavioural process, or other variables mediate this linkage. Fourth, our sample included elderly spousally bereaved people; the degree to which findings generalize to other bereaved groups remains to be examined. Fifth, although several statistically significant associations between SOC factors and outcomes were observed, these were generally quite small. Pending future research in this area, we should remain cautious in drawing conclusions about the impact of SOC in grief.

Notwithstanding these considerations, this is one of the, if not *the* first study showing that, in the face of losing a spouse at older age, SOC, and particularly its element of meaningfulness, may function as a buffer for the psychological impact of bereavement. More research is needed to examine the role of SOC in recovery from loss across different subgroups of bereaved people and variables mediating the impact SOC. Studying SOC and other factors protecting against negative health outcomes of bereavement seems all the more pertinent now that PGD is included in the main bodies of both the ICD‐11 (WHO, 2019) and DSM‐5‐TR (APA, 2022). If future research supports that SOC positively affects recovery from loss, some practical implications for supporting bereaved people may be considered. For instance, helping people to see the world as more comprehensible, manageable, and—particularly—more meaningful, and empowering them to identify resources needed to adjust may be important targets for interventions and health promotion activities (Super et al., 2016). Psychological interventions tailored specifically for recognizing and increasing a sense of meaning with life would, thus, be called for. Inspiration for these types of interventions may be gained from the perspective of positive psychology (Seligman & Csikszentmihalyi, 2000), narrative psychology (Nolen‐Hoeksema, 2000), and third wave cognitive behavioural psychology such as compassion focused therapy (Gilbert, 2014) or mindfulness based cognitive therapy (Segal et al., 2013).

## CONFLICT OF INTEREST

The authors declare to have no conflict of interest.

## Supporting information


**Table S1.** Factor loadings of the three‐factor model of the SOC‐13
**Table S2.** Summary of regression analyses with comprehensibility/manageability and meaningfulness predicting concurrent prolonged grief, depression, and satisfaction with life
**Table S3.** Summary of regression analyses with comprehensibility/manageability and meaningfulness predicting prolonged grief, depression, and satisfaction with life at W2
**Table S4.** Summary of regression analyses with comprehensibility/manageability and meaningfulness predicting prolonged grief, depression, and satisfaction with life at W3

## Data Availability

The data that support the findings of this study are available from the corresponding author (PAB) upon reasonable request.

## ENDNOTE

^1^
The ICG developed by Prigerson et al. (1995) includes 19 items. Several shortened versions have been used in consecutive studies, including this 15 item version. It was obtained from Prigerson at the start of the project and includes all 19 items except those representing feeling envious of others who have not suffered loss, survivor guilt, seeing the deceased, and hearing the deceased. In a study on the distinctiveness of disordered grief and posttraumatic stress, based on a sample that was different from the sample of this study, O'Connor et al. (2010) reported findings attesting to the internal consistency and construct validity of this 15 item ICG.
