# Supplementary material for: Is a sense of coherence associated with prolonged grief, depression, and satisfaction with life after bereavement? A longitudinal study
Source: Clin Psychol Psychother. 2022 Aug 16;29(5):1599–610. doi: 10.1002/cpp.2774 (PMC9804467; doi:10.1002/cpp.2774)
Supplement: Supplementary file 1 — Table S1. Factor loadings of the three‐factor model of the SOC‐13 Table S2. Summary of regression analyses with comprehensibility/manageability and meaningfulness predicting concurrent prolonged grief, depression, and satisfaction with life Table S3. Summary of regression analyses with comprehensibility/manageability and meaningfulness predicting prolonged grief, depression, and satisfaction with life at W2 Table S4. Summary of regression analyses with comprehensibility/manageability and meaningfulness predicting prolonged grief, depression, and satisfaction with life at W3 [file CPP-29-1599-s001.docx]

**Supplementary Table 1**

Factor loadings of the three-factor model of the SOC-13

|  | Comprehensibility |  |  |  |
| --- | --- | --- | --- | --- |
| 1 | Has it happened in the past that you were surprised by the behaviour of people whom you thought you knew well? | 0.398 |  |  |
| 2 | Do you have the feeling that you are in an unfamiliar situation and don’t know what to do? | 0.612 |  |  |
| 3 | Do you have very mixed-up feelings and ideas? | 0.654 |  |  |
| 4 | Does it happen that you have feelings inside you would rather not feel? | 0.630 |  |  |
| 5 | When something happened, have you generally found that: (you overestimated or underestimated its importance – you saw things in the right proportion) | 0.570 |  |  |
|  | Manageability |  |  |  |
| 6 | Has it happened that people whom you counted on disappointed you? |  | 0.509 |  |
| 7 | Do you have the feeling that you’re being treated unfairly? |  | 0.531 |  |
| 8 | Many people – even those with a strong character – sometimes feel like sad sacks (losers) in certain situations. How often have you felt this way in the past? |  | 0.419 |  |
| 9 | How often do you have feelings that you’re not sure you can keep under control? |  | 0.662 |  |
|  | Meaningfulness |  |  |  |
| 10 | Do you have the feeling that you don’t really care about what goes on around you? |  |  | 0.570 |
| 11 | Until now your life has had: (no clear goals or purpose at all – very clear goals and purpose) |  |  | 0.509 |
| 12 | Doing the things you do every day is: (a source of deep pleasure and satisfaction – a source of pain and boredom) |  |  | 0.705 |
| 13 | How often do you have the feeling that there’s little meaning in the things you do in your daily life? |  |  | 0.779 |

Note. SOC = sense of coherence.

**Supplementary Table 2**

Summary of regression analyses with comprehensibility/manageability and meaningfulness predicting concurrent prolonged grief, depression, and satisfaction with life

|  | Model 1 | Model 2 | Model 3 |
| --- | --- | --- | --- |
| DV = Prolonged grief at Wave 1 |  |  |  |
| IV = Comprehensibility/manageability (Beta) | -.29*** | - | -.14† |
| Meaningfulness (Beta) | - | -.35*** | -.28** |
| F | 18.54 | 28.04 | 16.44 |
| DF | 1, 195 | 1, 196 | 2, 193 |
| p | < .001 | < .001 | < .001 |
| Adjusted R^2^ | .082 | .121 | .137 |
|  |  |  |  |
| DV = Depression at Wave 1 |  |  |  |
| IV = Comprehensibility/manageability (Beta) | -.56*** | - | -.35*** |
| Meaningfulness (Beta) | - | -.58*** | -.38***  38*** |
| F | 97.24 | 111.71 | 77.98 |
| DF | 1, 209 | 1, 210 | 2, 207 |
| p | <.001 | <.001 | <.001 |
| Adjusted R^2^ | .31 | .34 | .42 |
|  |  |  |  |
| DV = Satisfaction with life at Wave 1 |  |  |  |
| IV = Comprehensibility/manageability (Beta) | .47*** | - | .15* |
| Meaningfulness (Beta) | - | .64*** | .55*** |
| F | 60.84 | 149.76 | 78.94 |
| DF | 1, 209 | 1, 211 | 2, 207 |
| p | <.001 | <.001 | <.001 |
| Adjusted R^2^ | .222 | .412 | .427 |

Note. DV = Dependent variable. IV = independent variable.

* p < .05. ** p < .01. *** p < .001.

**Supplementary Table 3**

Summary of regression analyses with comprehensibility/manageability and meaningfulness predicting prolonged grief, depression, and satisfaction with life at W2

|  | Model 1 | Model 2 | Model 3 |
| --- | --- | --- | --- |
| DV = W2 Prolonged grief |  |  |  |
| IV = W1 Prolonged grief (Beta) | .87*** | .85*** | .85*** |
| Comprehensibility/manageability (Beta) | < -.01 | - | .05 |
| Meaningfulness (Beta) | - | -.05 | -.07 |
| F | 239.34 | 236.66 | 155.86 |
| DF | 2,151 | 2, 151 | 3, 149 |
| p | <.001 | < .001 | <.001 |
| Adjusted R^2^ | .757 | .755 | .753 |
|  |  |  |  |
| DV = W2 Depression |  |  |  |
| IV = W1 Depression (Beta) | .74*** | .70*** | .69*** |
| Comprehensibility/manageability (Beta) | -.06 | - | -.03 |
| Meaningfulness (Beta) | - | -.11† | -.10 |
| F | 115.88 | 114.19 | 75.16 |
| DF | 2, 164 | 2, 164 | 3, 162 |
| p | <.001 | <.001 | <.001 |
| Adjusted R^2^ | .581 | .577 | .574 |
|  |  |  |  |
| DV = W2 Satisfaction with life |  |  |  |
| IV = W1 Satisfaction with life (Beta) | .63*** | .56*** | .55*** |
| Comprehensibility/manageability (Beta) | .17** | - | .08 |
| Meaningfulness (Beta) | - | .25*** | .22** |
| F | 87.33 | 100.41 | 66.73 |
| DF | 2, 163 | 2, 163 | 3, 161 |
| p | <.001 | <.001 | <.001 |
| Adjusted R^2^ | .511 | .546 | .546 |

Note. DV = Dependent variable. IV = Independent variable. W1 = Wave 1. W2 = W2. W3 = Wave 3. * † p < .10. p < .05. ** p < .01. *** p < .001.

**Supplementary Table 4**

Summary of regression analyses with comprehensibility/manageability and meaningfulness predicting prolonged grief, depression, and satisfaction with life at W3

|  | Model 1 | Model 2 | Model 3 |
| --- | --- | --- | --- |
| DV = W3 Prolonged grief |  |  |  |
| IV = W1 Prolonged grief (Beta) | .83*** | .80*** | .81*** |
| Comprehensibility/manageability (Beta) | -.04 | - | -.09 |
| Meaningfulness (Beta) | - | -.05 | -.10† |
| F | 159.54 | 156.87 | 104.94 |
| DF | 2, 151 | 2, 151 | 3, 149 |
| p | <.001 | < .001 | <.001 |
| Adjusted R^2^ | .675 | .671 | .672 |
|  |  |  |  |
| DV = W3 Depression |  |  |  |
| IV = W1 Depression (Beta) | .74*** | .69*** | .68*** |
| Comprehensibility/manageability (Beta) | -.06 | - | -.02 |
| Meaningfulness (Beta) | - | -.13* | -.13* |
| F | 113.43 | 116.33 | 76.48 |
| DF | 2, 158 | 2, 158 | 3, 156 |
| p | <.001 | <.001 | <.001 |
| Adjusted R^2^ | .584 | .590 | .588 |
|  |  |  |  |
| DV = W3 Satisfaction with life |  |  |  |
| IV = W1 Satisfaction with life (Beta) | .61*** | .53*** | .50*** |
| Comprehensibility/manageability (Beta) | .17** | - | .06 |
| Meaningfulness (Beta) | - | .28*** | .26*** |
| F | 77.29 | 97.06 | 64.18 |
| DF | 2, 157 | 2, 157 | 3, 155 |
| p | <.001 | <.001 | <.001 |
| Adjusted R^2^ | .490 | .547 | .545 |

Note. DV = Dependent variable. IV = Independent variable. W1 = Wave 1. W2 = W2. W3 = Wave 3. * p < .05. ** p < .01. *** p < .001.
